# A horizon scanning exercise to explore retention policies for international and minoritised NHS Trust staff in England: what are the current pledges and where are the gaps?

**DOI:** 10.1186/s12913-025-13348-7

**Published:** 2025-10-09

**Authors:** Rebecca Moss, Carol Rivas, Adam Mann, Manish Pareek, Katherine Woolf, I-CARE Study Collaborative Group, Carol Woodhams, Sorin Krammer, Anna L. Guyatt, Asta Medisauskaite, Srinivasa Vittal Katikireddi, Padmasayee Papineni, Susie Lagrata, Mehrunisha Suleman, Asad Masood, Luke Bryant

**Affiliations:** 1 University College London Medical School, London, UK; 2 Imperial College London Faculty of Medicine, London, UK; 3 Department of Respiratory Sciences, University of Leicester, Leicester, UK; 4 Department of Population Health Sciences, University of Leicester, Leicester, UK; 5 Department of Infection and HIV Medicine, University Hospitals of Leicester NHS Trust, Leicester, UK; 6 Development Centre for Population Health, University of Leicester, Leicester, UK; 7 Leicester NIHR Biomedical Research Centre, Leicester, UK; 8 NIHR Applied Research Collaboration East Midlands, Leicester, UK

**Keywords:** Staff retention, Policies, Minoritised, International, NHS Trusts, England

## Abstract

**Background:**

The NHS has pledged to reduce staff attrition to address its widening workforce gap, which has been exacerbated by understaffing and the COVID-19 pandemic. Evidence suggests that staff from some minoritised groups may be at greater risk of attrition post-pandemic. However, a gap in the literature means there is no clear overview of current organisation-level NHS staff retention policies aimed at addressing this problem. We sought to address this with a horizon scanning exercise undertaken as part of a wider study aiming to support the development of policies to improve retention of NHS staff from minoritised groups. The study draws on three key occupational psychology theories, each of which provides different insight into the mechanisms underlying staff turnover and its major contributing factors of sickness and satisfaction.

**Methods:**

The sampling frame was all 215 NHS Trusts in England. Within each region we sampled NHS Trusts with numbers of minoritised or international staff above the regional median and rates of staff retention (stability) below the regional median, based on publicly available NHS statistics. We sourced retention policies directly from Trust staff or their public facing websites. We also sourced policy documents that focused on induction or Equality, Diversity and Inclusion in case they contained relevant content. We extracted and analysed the contents of documents based on preselected aspects of work known to influence staff attrition, developed from the underpinning theories. We used Framework Analysis to make comparisons within and between documents and Trusts.

**Results:**

Documents were sent to us by 13% of the sampled Trusts. We found other documents by searching Trust websites. We obtained and screened 128 documents from 56 Trusts and analysed 99 that met our inclusion criteria. Trusts were considering staff retention, and many of them had actionable strategies. Most Trusts had enshrined some workplace matters in policy that may be particularly relevant to international and minoritised staff. Examples were anti-discrimination and bullying procedures and inclusive line management which may improve retention by reducing job demands and work stress. However, other matters that may influence retention of international and minoritised staff were rarely addressed. Notably lacking was consideration of the range of factors that contribute to creating and maintaining a sense of belonging and trust such as provided by induction, onboarding and effective staff networks.

**Conclusion:**

To our knowledge this is the first study to collect and analyse current Trust level NHS staff retention policies in England. The results are important because they reveal that while some factors that may influence retention of minoritised and international staff were comprehensively addressed by the sampled Trusts’ policy documents, many others were absent. The documents are reflective of organisations with social and institutional awareness of staff-wide retention drivers but not necessarily cognisant of specific additional drivers of attrition for international and minoritised staff. We recommend that policy makers reframe generic statements regarding attrition and retention as concrete procedures with specified measurable outcomes. Our findings will now contribute to the co-creation of a suite of retention policy interventions in collaboration with our stakeholder groups.

**Supplementary Information:**

The online version contains supplementary material available at 10.1186/s12913-025-13348-7.

## Background

The 2023 NHS Long Term Workforce Plan (LTWP) pledged to improve retention and ensure 130,000 fewer staff leave the NHS over the next 15 years by improving culture, leadership and wellbeing [1]. The LTWP acknowledges that, without concerted and immediate action, the NHS will face a workforce gap of more than 260,000–360,000 staff by 2036/37 [1]. Workload pressures, poor work-life balance, mental ill-health, and poor development opportunities are major reasons why healthcare workers (HCWs) leave [2, 3] and these problems have been exacerbated by understaffing and the pandemic [4, 5].

Ethnic minority HCWs (self-categorising as Black, Asian, Mixed, Other) comprise 24% of all NHS staff, including 42% of all doctors [6] and over 90% of nurses in the lowest pay grade [7]. Staff from minoritised groups are at greater risk of harassment, low pay and poor career progression [6, 8, 9] and they have been disproportionately negatively affected by the pandemic [10, 11]. In a 2022 survey of NHS leaders from ethnic minority groups, over half said discrimination made them want to leave [12]. Racial discrimination causes poor physical and mental health [13] and may increase sickness absence, which strongly predicts attrition [14]. Taken together, this evidence strongly suggests that staff from minoritised groups may be at greater risk of attrition post-pandemic, as indicated by the findings of the United Kingdom Research study into Ethnicity And COVID-19 outcomes in Healthcare workers (UK-REACH) study on the impact of Covid-19 on UK HCWs from ethnic minority groups [15].

The InCreAsing REtention of healthcare staff from Ethnic minority groups (I-CARE) study (for overview see Appendix 1) grew from UK-REACH, and its aim is to improve retention of minoritised staff groups by informing NHS retention policies. However, there is currently no clear overview of Trust level NHS staff retention policies aimed at addressing turnover of minoritised and international staff.

The aim of the I-CARE study phase we report here was to address this gap in the literature with a horizon scanning exercise. The objective was to extract and analyse the document contents drawing on three major theories of staff retention and its prime contributing factors, sickness and job satisfaction (job embeddedness theory [16], job demands-resources model [17], and self-determination theory [18, 19]) in order to a) identify existing policy approaches to improving retention of minoritised staff and b) identify potential policy gaps.

May [20] argues that ‘documents, read as the sedimentations of social practices, have the potential to inform and structure the decisions which people make on a daily and longer-term basis: they also constitute particular readings of social events’ (p176) [20]. Thus policy documents represent more than passive neutral information and instead function as embodiments of social and institutional behaviour [21], in that their textual communicative practices constitute part of the reality of working for them. Document analysis of NHS Trusts policies can provide insight into the existing practices that might affect retention of staff from minoritised groups and demonstrate where there are policy gaps.

## Methods

### Study design

Desk-based horizon scanning exercise and document content analysis.

### Sampling framework

We developed a sampling framework to include NHS Trusts with high numbers of minoritised staff and low rates of staff retention as these were more likely to be revealing of unmet needs. Within each NHS region (East of England, London, Midlands, Northeast and Yorkshire, Northwest, Southeast, Southwest), we selected Trusts that had staff stability below the regional median and proportions of staff from ethnic minority groups above the regional median. We used regional medians to ensure coverage across England as otherwise the data would be dominated by London Trusts given the concentration of migrant populations there. We obtained staff stability index numbers from the Monthly Turnover from Organisation by Staff Group figures published by NHS England [22]. The stability index is calculated by dividing the number of staff at the start of a period by the number of staff who remain at the end of that period. We obtained trust proportions of ethnic minority staff based on the NHS Workforce Race Equality Standard Report [23]. We defined staff as ethnic minority if they self-categorised as Black, Asian, Mixed, Other in the NHS staff survey. Within the main sampling frame, we included a range of Trust types to ensure representation from acute, large, small, teaching, community, ambulance and mental health Trusts.

### Data collection

A Chief Executive Officer at one of the sampled Trusts who was a personal contact of the authors emailed the leadership teams at the remaining Trusts, inviting them to email the I-CARE study team with any of the following documents:

- Current retention policy documents specifically for international and/or ethnic minority staff from any professional/occupational groups,
- Human resources policies such pertaining to Equality, Diversity and Inclusion (EDI), wellbeing or induction that may have been aimed, wholly or in part, to improve retention,
- Activities undertaken as part of the People Promise Exemplars programme (in which a group of acute, community and mental health providers are working with NHS England to implement the NHS Our People Promise [24]. which aims to improve staff wellbeing)
- Evaluations of retention activities.

We offered NHS Trust leadership teams the opportunity to discuss the I-CARE study further with a member of the research team if they wished to do so. Where Trusts did not provide documents, BM searched Trust websites for relevant documents in the public domain. Documents were included if they made any reference to retention, induction or EDI and excluded if they did not refer to any of these three areas. In most instances exclusion occurred where Trusts had sent us a large volume of documents in case they were of relevance. To avoid inadvertently missing relevant information, documents referred to as plans, strategies, visions, promises, aims or proposals were included, besides those explicitly labelled policies.

### Procedure

We adopted Dalgliesh et al.’s [25] READ approach to document analysis: (1) read materials, (2) extract data, (3) analyse data and (4) distil the findings. BM screened all documents for inclusion/exclusion. BM extracted all document data and AM extracted data from a subset of 20 randomly selected documents, using a structured form developed collaboratively with the wider I-CARE team and based on the three theories:

1. Job embeddedness theory of retention and turnover [16];
2. Job demands-resources model of psychosocial work conditions and staff wellbeing or illness [17];
3. Self-determination theory of motivation [18] applied to work engagement and performance [19].

Job embeddedness theory focuses on contextual factors that encourage people to stay in a job, such as links to other individuals, activities and communities, how easy or difficult it would be to break these links, and how well their job fits into other aspects of their lives [16]. Job demands-resources theory describes a balance model of both negative and positive indicators of employee wellbeing. Negative indicators (demands) can include high job stress, unfavourable physical environment and challenging interpersonal encounters; conversely, positive indicators (resources) can include opportunities for career advancement, favourable team climate and involvement in decision making [17]. Finally, self-determination theory is based on three basic human psychological needs: autonomy, competence and relatedness – feeling connected and meaningfully involved. When these needs are met, they increase motivation, but if they are not, they reduce it, and have a deleterious effect on wellbeing [18, 19].

The extraction form used categories developed from these theories and also allowed for additional more inductive category suggestions, though in the event, none were added. The three main extraction categories were promotion and progression, recognition and rewards, and staff training and development, with subsequent cascades of questions capturing fine-grained detail within each category. The survey logic of the data extraction form meant that if a category appeared in a document, we were able to capture further detail in subcategories, and if a category did not appear the survey automatically directed us to the next section of the form. The full data extraction question list can be found in Appendix 2. We hosted the data extraction form on RedCap (www.project-redcap.org) [26], a secure web application for building and managing online surveys and databases. We chose RedCap rather than a more conventional Computer-Assisted Qualitative Data Analysis Software (CAQDAS) [27] approach after considering the concise nature of the text in the policy documents. At the beginning of data extraction, CR, who entered the form onto RedCap, provided BM and AM with written training instructions, following which CR, BM and AM then each independently reviewed three initial documents which CR checked for consistency. This exercise also served to pilot the form design, leading to improvements in wording and logic flow.

### Analysis

We exported extracted data from RedCap in .csv format in a matrix, which we imported into NVivo software [.^28^]. We used Framework Analysis [29] to make comparisons within and between documents and Trusts. Analysis was mainly deductive and descriptive rather than conceptual so that findings would inform other work packages in the wider I-CARE study. Initially, Framework matrix columns corresponded to the categories used in the extraction sheet and rows represented the individual documents, then rows across each Trust were amalgamated. We counted the number of statements appearing under each category and sub-category. We then cross-referenced the frequencies of each sub-category against each of the three retention-related theories, in order to provide a visual map of the coverage of retention-related themes across the three theories.

To assess the quality of the policy statements within documents, we classified document contents using a Red-Amber-Green (RAG) system, where red indicated absence/no information on a category, amber indicated that a category was mentioned but lacked explicit or measurable targets as to how it may be achieved, and green denoted policy statements that were linked with actionable, specific objectives. Individual statements within documents were rated as Red/Amber/Green. A single Trust could therefore have several documents, each of which contained Red, Amber or Green-rated statements. We counted the number of green, amber and red statements and used this to estimate the frequency of high-quality (green) policy statements for each category.

### Ethical approval

This phase of the I-Care study was a paper-based documentary analysis of hospital policies with no research participants or human material. The University of Leicester sponsor confirmed that ethics approval and consent procedures were therefore not required.

### Patient and Public Involvement and Engagement (PPIE)

We held Patient and Public Involvement and Engagement (PPIE) Workshops with the I-CARE Patient Involvement Panel (PIP), the Professional Expert Panel (PEP) and the Stakeholder Advisory Group (STAG). in which we shared examples of retention documents and preliminary content analysis and sought their comments on the documents’ contents and style. PPIE workshops are integrated throughout the I-CARE study and will eventually inform a programme theory with synthesised research findings from all the I-CARE work packages. A suite of theory-based policy interventions will be co-designed with the PPIE and other stakeholders.

## Results

### Document retrieval

**Fig. 1:**
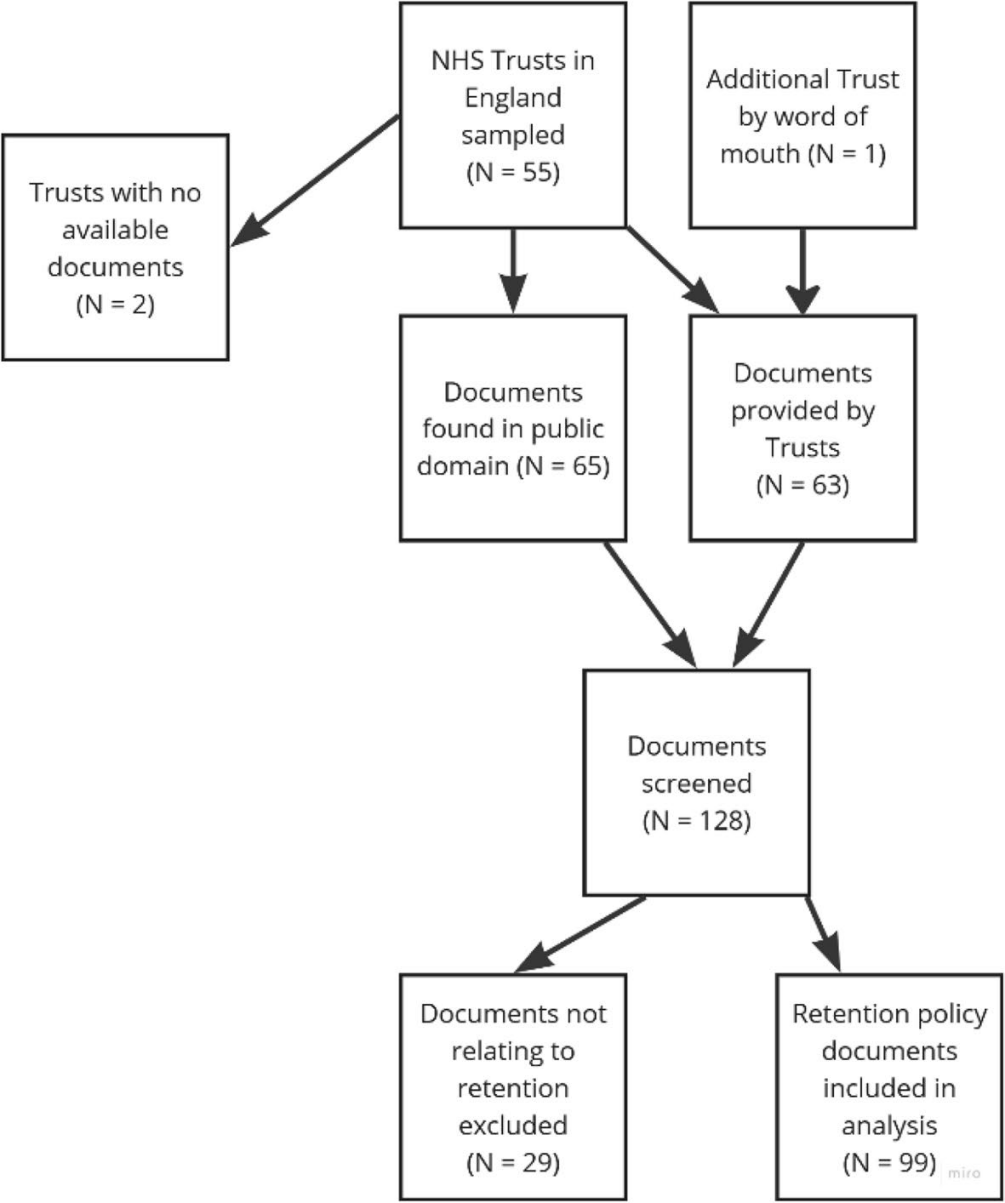
Retention policy document flowchart

We included in the analysis 99 documents obtained from 56 NHS Trusts in England (see Fig. 1 for flow diagram). There were 55 NHS Trusts in the original sample and one additional Trust learned of the I-CARE study by word of mouth and volunteered documents, which we included because another Trust in their region did not volunteer documents. Of these 56 Trusts, seven (13%) provided policy documents. Of the 49 (87%) Trusts that did not provide documents, three responded that they did not have specific retention policies and referred us to their all-staff documents and Trust strategies; eight sent cordial responses endorsing the study but did not provide specific documents and the remaining 38 Trusts did not respond to our request for information. We obtained documents for 47 of these 49 Trusts via web searches. Locations of documents varied according to website designs but were most often found within their ‘About Us’ or their Equality, Diversity and Inclusion sections. No information was available in the public domain for the remaining two. We reviewed 128 documents; we excluded 29 as not relevant to staff retention and included the remaining 99 documents in the analysis.

### Frequency of categories across all policy documents

**Fig. 2:**
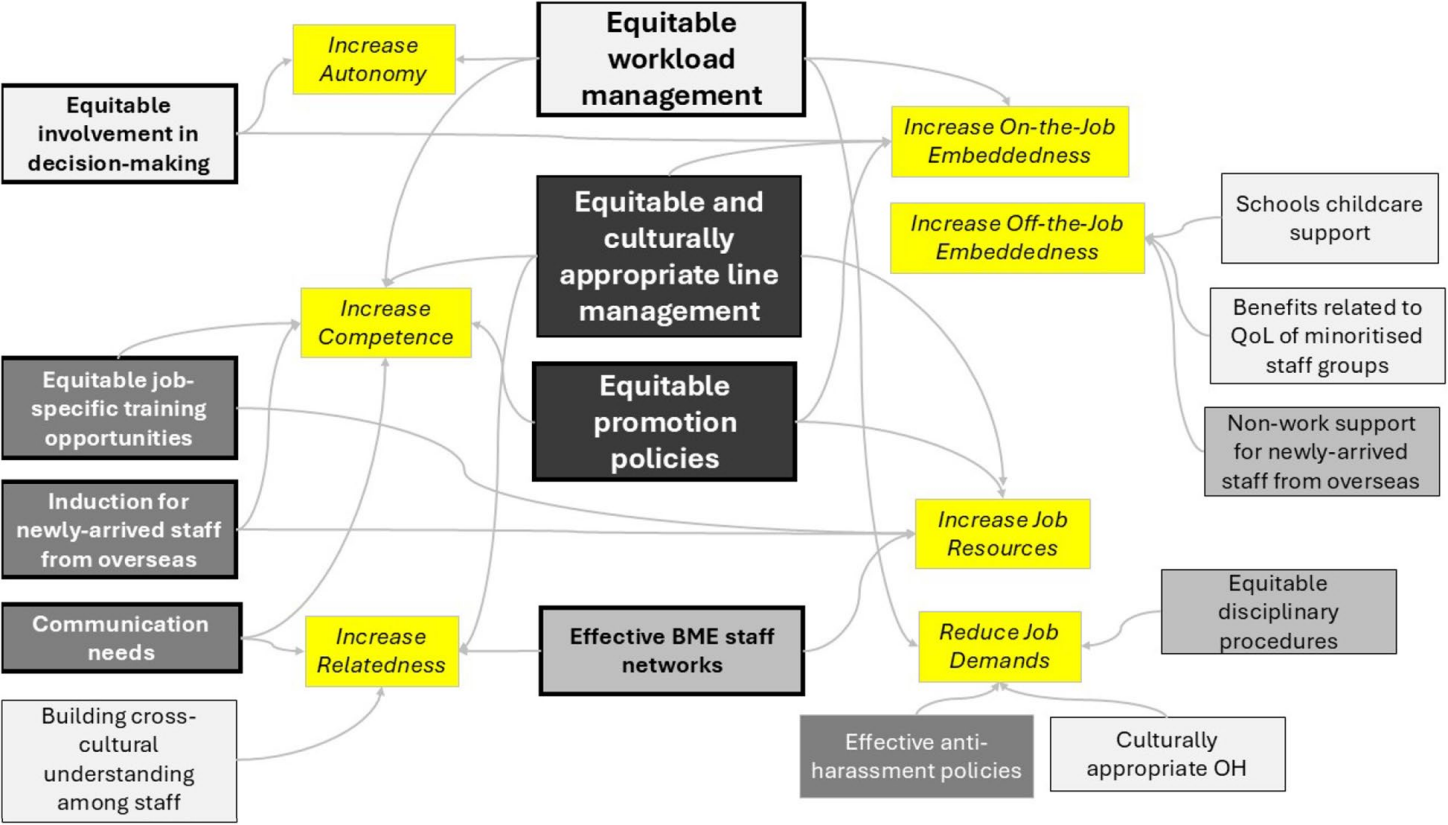
Category mentions relating to the three retention-related theories by frequency of occurrence

Figure 2 allows examination of the frequency of categories mentioned in all policy documents in relation to each of the three retention-related theories. In the figure, darker shading indicates that a category was mentioned more frequently within the policy documents, and larger text size indicates that the categories were relevant to multiple theories. The three theories in yellow are self-determination (comprising autonomy, competence and relatedness), job-demands and resources, and job embeddedness (off-the-job and on-the-job).

The figure demonstrates that two of the most frequently mentioned categories ‘equitable and culturally appropriate line management’ and ‘equitable promotion policies’, were those that related to all three theories. By contrast, the category of ‘equitable workload management’ was also considered to be related to all three theories but was relatively infrequently mentioned.

Other categories, such as ‘equitable job-specific training opportunities’, ‘induction for newly-arrived staff from overseas’, ‘communication needs’, and ‘effective anti-harassment policies’ received fairly frequent mention, but these were considered to relate to fewer theories. Policies related to quality of life in non-work life (embeddedness), culturally appropriate occupational health provision (demands-resources) and building of staff cross-cultural trust and understanding (self-determination of motivation) were infrequently mentioned.

### Frequency of green-rated actionable categories across all policy documents

**Fig. 3:**
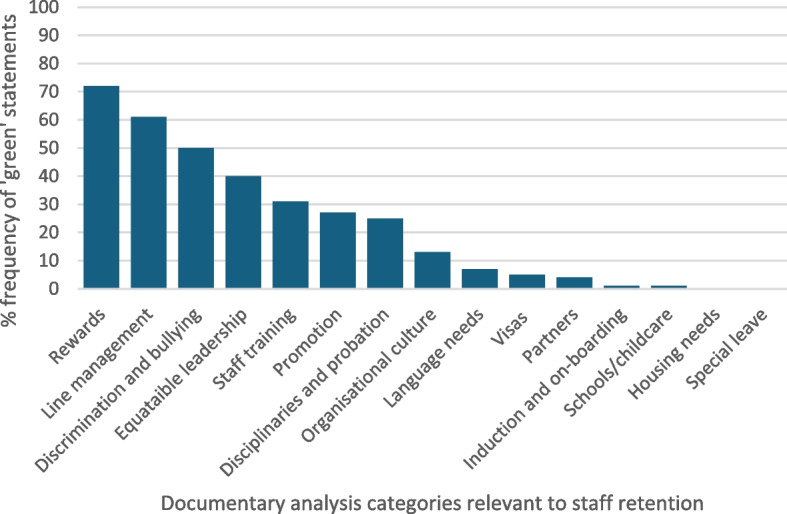
Category coverage in documents with green-rated statements

Figure 3 shows the frequency of categories across all documents that we considered to have been discussed well within policy documents (i.e. linked to actionable and specific objectives) and thus we coded green in the RAG system. It shows a clear disparity: while some categories were frequently discussed well in policy documents e.g. rewards, line management, others were rarely or never discussed well e.g. induction/onboarding, housing needs.

For example, a green-rated statement addressed anti-discrimination processes and appropriate line management as follows:

‘The [Trust] Strategy emphasises inclusive line management processes, particularly addressing ethnicity, race, and migrant status. Managers receive training in unconscious bias and cultural competency to ensure fair treatment and support for all staff, especially those from BAME [*sic*; Black, Asian and Minority Ethnic] backgrounds. The strategy promotes gender equity, recognising contributions from staff of all ages, and ensures fair treatment across all levels of seniority and occupational roles. It also includes provisions for reasonable adjustments for staff with disabilities and respects diverse religious practices.’

An example of a green-rated statement that foregrounded minoritised and international staff in the Trust’s rewards and recognition policy was as follows:

‘The strategy focuses on ensuring that all staff, irrespective of ethnicity, race, or migrant status, feel appreciated and valued. The Trust implements initiatives like the monthly “Living Our Values” awards to celebrate the contributions of its diverse workforce … Other intersecting factors, such as socio-economic status, are also considered to ensure a fair and comprehensive recognition system.’

There were pockets of excellent practice that revealed careful consideration of international and minoritised staffs’ specific needs. For example, one document from a large Trust in the London region had produced a 52-page information booklet for International Medical Graduates, with a wealth of welcoming and orienting material including sections on local food, weather, currency, a list of British idioms and colloquialisms and how to register with a GP and dentist, all of which would function to increase off-the-job embeddedness. A different London Trust described offering staff 12 sessions of reciprocal mentoring, designed to understand the experiences of staff described as from the global majority, and pledged to use this understanding to improve staff experience of clinical services; these are both clear means of increasing on-the-job embeddedness.

However, these instances were rare, and across the whole range of documents matters of potential importance to international and minoritised staff were seldom addressed. Notably lacking was the range of factors which may influence settling into a new environment, both in and beyond the workplace, including induction, onboarding, visas, housing, schools/childcare, partners, language needs and special leave. Instead, many of the documents focused on generic EDI statements addressing the nine protected characteristics of the UK Equality Act 2010 [30] for all Trust staff and did not go beyond these to address the specific needs of their international and minoritised staff. We did not compare Trusts by type or by region as the numbers were too small to break down further; for example, there were only four mental health Trusts in the sample, three of which were in the London region.

In Table 1, we provide further information about the number of Trusts that mentioned each category within their documents, whether rated red, amber or green. We also provide one green-rated and one amber-rated example for each category.

**Table 1:** Examples of green and amber rated statements for each category theme

Category theme	Number (percentage) of Trusts	Green example	Amber example
Recognition and rewards	13 (24%)	The rewards and recognition policy within the strategy focuses on ensuring that all staff, irrespective of ethnicity, race, or migrant status, feel appreciated and valued. The Trust implements initiatives like the monthly “Living Our Values” awards to celebrate the contributions of its diverse workforce. Gender equity is emphasized by designing inclusive and equitable recognition programs that address any disparities. Age diversity is respected, with initiatives that celebrate contributions from employees of all ages. Recognition is distributed fairly across different levels of seniority, ensuring that both junior and senior staff feel valued. Occupational role diversity is considered by extending recognition to non-clinical staff. The Trust is committed to equitable recognition for employees with disabilities and respects religious diversity by ensuring inclusive recognition practices. Other intersecting factors, such as socio-economic status, are also considered to ensure a fair and comprehensive recognition system	We will focus our efforts to provide a comprehensive reward and recognition package to support staff retention … we will provide a comprehensive reward and recognition package to retain and develop a highly-skilled and flexible workforce
Line management and appraisal	17 (31%)	The line management policy aims to enhance the effectiveness and inclusivity of management practices within the organisation. It includes providing training for managers to support values-based appraisals and wellbeing conversations, ensuring they can give constructive and compassionate feedback. The policy also promotes the use of mentoring programmes to help managers listen to and support diverse voices. Additionally, line managers are encouraged to adopt flexible working policies and participate in leadership development programmes to align with the organisation’s inclusive and supportive culture. The overall goal is to equip managers with the skills and tools needed to effectively lead and support their teams	We will also ensure that all our people have a meaningful appraisal that includes wellbeing and career development conversations and we will continue to enhance the staff facilities that are on our sites
Discrimination, harassment and bullying	23 (42%)	The discrimination, harassment, and bullying policy at [Trust] aims to create a safe and inclusive workplace by eliminating all forms of discrimination, harassment, and bullying. Key initiatives include the launch of a Zero Acceptance Policy and a high-profile ‘We Do Not Accept’ campaign to tackle discrimination, racism, bullying, harassment, violence, aggression, and abuse. The policy also emphasises the importance of fostering a culture of kindness and respect, with interventions like Civility and Respect workshops. The Trust is committed to improving staff engagement and reducing incidents of bullying and harassment, as measured by staff surveys and regular feedback mechanisms	The People Strategy will enable the Trust to work towards our ambition of becoming an actively anti racist organisation and remove systemic racism and discrimination which may exist. We will improve staff experience concerning inclusion and representation across the Trust and eliminate discrimination
Equitable leadership	19 (35%)	The equitable organisational leadership policy at [Trust] aims to foster an inclusive and diverse leadership environment that supports equitable career progression for all staff. Key initiatives include the implementation of the ‘Mend the Gap’ self-assessment actions to support female medical staff from minority ethnic backgrounds in advancing to leadership roles such as Speciality Leads and Clinical Directors. Additionally, the policy includes launching a new digital coaching platform to improve access to mentors and coaches, developing a comprehensive talent management strategy by December 2025, and establishing formal programmes of career coaching and mentorship targeting staff on apprenticeship programmes or those wishing to develop further. The policy specifically addresses the needs of staff from diverse ethnic backgrounds and migrant statuses by implementing measures to combat racism and ensure equitable treatment for all	We will develop and improve our equality performance and increase diversity within our board and senior leadership teams. We aim to overhaul our recruitment, promotion and flexible working practices, increase leadership diversity across the organisation and widen employment opportunities to support our community
Staff training and development	23 (42%)	The staff training and development policy aims to build a knowledgeable and culturally aware workforce equipped to serve diverse communities. Key initiatives include providing cultural awareness training, organising “Let’s Talk” sessions and EDI events, and distributing EDI bulletins for staff unable to attend training sessions. The policy also emphasises the importance of involving staff in the development and implementation of action plans through team meetings and consultations. Additionally, it focuses on creating resources, such as directories and newsletters, to support ongoing learning and awareness about diversity, equity, and inclusion issues	The Trust is committed to ensuring that all employees are supported in developing the skills and abilities they require to carry out their current and any likely future role in the organisation. Use of appraisal and equality of access for all employees to both training and development opportunities is key and will be subject to regular monitoring
Promotion and progression	22 (40%)	The policy acknowledges the importance of addressing the needs of staff from diverse ethnic backgrounds and migrant statuses. Specific actions include supporting internationally educated staff by providing additional support to ensure their successful integration and career progression. The policy outlines actions to support under-represented groups, such as the ‘Mend the Gap’ initiative aimed at supporting female medical staff, particularly from minority ethnic backgrounds, to progress into leadership roles like Speciality Leads and Clinical Directors	We will make sure that everyone has opportunities to develop in their career, whatever your ambitions are. We will be innovative in how we help you learn, develop and grow in our Trust. We will remove barriers to progression
Disciplinaries and probation	10 (18%)	In relation to ethnicity, race, and migrant status, the Trust’s disciplinary and probation policies are integrated with its broader commitment to equality, diversity, and inclusion. This ensures that all staff, regardless of their background, are treated equitably throughout the disciplinary and probation processes. The Trust’s initiatives like the ‘Removing the Barriers Programme’ support the fair treatment of Black, Asian, and Minority Ethnic staff by promoting diversity in leadership and ensuring that all disciplinary actions are free from bias. Intersecting factors such as gender, age, seniority, occupational role, disability, and religion are also considered, with specific measures in place to address the unique challenges faced by different groups. This comprehensive approach ensures a fair, supportive, and inclusive environment for all employees	Acts will be dealt with as misconduct under the organisation’s grievance and/or disciplinary procedures, and any appropriate action will be taken
Organisational culture	22 (40%)	[Service] launched ‘Kindness Counts’ Campaign to foster a culture of kindness and mutual respect in [hospital] and across the organisation. [Service] developed a campaign themed ‘The Culture We Desire’ and to promote National Bullying Prevention Month and National Anti-Bullying Awareness Week. Launched civility and respect videos to bring together all the workforce initiatives that link to supporting a positive and inclusive culture at work, which appears to have been well received by staff and has had 2,232 views to date	The document highlights a commitment to fostering an inclusive organisational culture, emphasising the importance of cultural sensitivity and collective responsibility in shaping a welcoming environment. This approach is intended to ensure that every team member, regardless of their ethnic or racial background, feels valued and respected. The organisation aims to integrate these principles into their daily operations and strategic objectives, promoting a culture where diversity is recognised as a strength that enhances their operational effectiveness
Induction and onboarding (including reference to visas)	15 (27%)	In order to support effective retention rates at the Trust, [name] is committed to providing an inclusive and welcoming environment for new employees. At the start of an employee’s employment into the Trust, they will be invited to attend the Trust Corporate Induction and the line manager should also arrange a local induction for the new starter. The Trust induction is a one-day programme. Managers must ensure that they provide a robust local induction for their new employee, ensuring that all systems and processes are explained in the department for example payroll, local health and safety policies, Annual Leave, Time off In Lieu, Flexible Working policies and access to relevant IT systems. For the local induction it is best practice for line managers to arrange a “getting to know you” induction plan which should include a list of key contacts and individuals that the individual will need to meet in order to orientate them into their new role. Where possible the getting to know you orientation/induction plan should involve a meeting with the Head of Service and Director where appropriate and should last for at least two weeks to allow the employee to settle into their new role	We will implement a comprehensive induction, onboarding and development programme for internationally-recruited staff, ensuring that, wherever in the world our people join us from, they are welcomed with an inclusive, equitable and comprehensive onboarding programme, sustained care and development

## Discussion

We have undertaken the first horizon scanning exercise in England examining current retention policies at a Trust level in NHS Trusts most in need of effective procedures, namely those with high numbers of minoritised staff and low rates of staff retention. The descriptive category findings from our analysis, developed from existing theory, revealed a nuanced and somewhat chequered picture in which some factors that may influence retention of minoritised and international staff were frequently and comprehensively addressed within policy documents, but many others were absent from policy. In particular, factors that contribute to creating and maintaining a sense of belonging and trust [31–33] were frequently overlooked.

Effective staff networks were rarely discussed, yet these offer opportunities to make links with other individuals and communities, potentially increasing on-the-job embeddedness, and have been shown to decrease the stress and isolation experienced by minoritised NHS staff in the wake of the pandemic [31]. NHS guidance [32] recommends a five-step induction for international medical graduates (doctors who achieved their primary medical qualification outside the UK), which covers welcome and pastoral care, professional practice induction, language and communication induction, IT systems induction, and specialty induction. Yet fewer than 5% of documents referred to the support HCWs relocating to the UK may need to help integrate into an unfamiliar healthcare system and a different cultural environment. Factors influencing work-life balance such as local amenities and leisure activities for new arrivals were entirely neglected yet knowledge of these could boost acculturation to their host country [33]. They are also areas with the potential to significantly improve off-the-job embeddedness, which could reduce work/life conflict and therefore reduce staff turnover [32]. Finally, culturally appropriate Occupational Health and reasonable adjustments, and psychological support were not mentioned in the retention policy documents, but these are known to be drivers in reducing sickness leave and in turn attrition [34, 35]. These findings suggest that while some elements of the three major theories of job retention were attended to, others were largely neglected.

From a document analysis perspective, the NHS Trust retention policy documents described here suggest organisations have social and institutional awareness of certain concepts and buzzwords that are namechecked on a staff-wide level, but they are not necessarily cognisant of the specific additional drivers of attrition for international and minoritised staff. Broad EDI statements or similar policy documents speak to aspects of the job demands and resources model, for example seeking to reduce unpleasant interpersonal encounters by eliminating discrimination, harassment and bullying. However, they may neglect more nuanced elements of embeddedness and personal autonomy, which may also be influential for job satisfaction and staff retention. Our horizon-scanning evidence suggests that many current official Trust level NHS retention policies overlook important potential drivers of attrition for international or minoritised staff. Further, when these drivers are discussed, they frequently lack actionable, measurable targets and explicit strategies to achieve these and instead are framed as somewhat vague good intentions. We recommend that policy makers reframe generic statements regarding attrition and retention as concrete procedures with specified measurable outcomes.

Our analysis has many strengths. Retention related documents were retrieved for a very high proportion of the Trusts sampled (96%). We analysed a comprehensive range of different types of documents including those pertaining to EDI, induction and staff wellbeing, besides those explicitly labelled as retention policies. We will issue a renewed call for documents at the midpoint of the I-CARE study and make every effort to encourage Trusts to actively provide these to us. We anticipate greater levels of engagement in the next wave of horizon scanning now that many of the sampled Trusts have committed to participating in other I-CARE work packages.

The study has some limitations. Most Trusts (87%) did not proactively send documents to us in this first stage of the horizon scanning exercise, and consequently these had to be found through Internet searches. One possible implication of this is that there may be documents in existence which are unknown to us. The RAG classification system is a subjective measure, and it is possible that some documents may be interpreted differently by other readers.

Findings from this stage of the I-CARE study indicated some gaps in NHS Trust retention policies and enabled us to gain a better on-the-ground understanding of the impacts of gaps in retention policies on minoritised and international NHS staff. The findings are being used to develop a theory of job retention in the form of a systems map and to acceptability testing of a suite of theory-based interventions designed to reduce staff attrition.

## Supplementary Information

Supplementary Material 1: Appendix 1. The wider I-CARE study.

Supplementary Material 2: Appendix 2. Redcap data extraction categories

Supplementary Material 3.

## Data Availability

Source documents which remain available in the public domain are listed in supplementary material with web links. Documents originally in the public domain but no longer visible on websites can be provided as PDF files on request. Documents provided directly by Trusts and not in the public domain are not available.
