# Supplementary material for: A horizon scanning exercise to explore retention policies for international and minoritised NHS Trust staff in England: what are the current pledges and where are the gaps?
Source: BMC Health Serv Res. 2025 Oct 9;25:1337. doi: 10.1186/s12913-025-13348-7 (PMC12509401; doi:10.1186/s12913-025-13348-7)
Supplement: Supplementary file 1 — Supplementary Material 1: Appendix 1. The wider I-CARE study. [file 12913_2025_13348_MOESM1_ESM.docx]

**Appendix 1: The wider I-CARE study**

InCreAsing REtention of healthcare staff from Ethnic minority groups (I-CARE) is a three-year study funded by the National Institute for Health and Care Research (NIHR). It is a mixed methods study comprised of five work packages (WPs):

WP1: Retention policy horizon scanning (reported here)

WP2: Retrospective analysis of NHS human resources record data from the Electronic Staff Record

WP3: Longitudinal analysis of six waves of healthcare worker questionnaire data

WP4: Qualitative analysis of interviews and focus groups with ethnically minoritised NHS staff and NHS managers

WP5: Development and refinement of a systems map, using synthesised findings from the previous four WPs to co-design and explore the acceptability of a suite of theory-based policy interventions.
