# Supplementary material for: A horizon scanning exercise to explore retention policies for international and minoritised NHS Trust staff in England: what are the current pledges and where are the gaps?
Source: BMC Health Serv Res. 2025 Oct 9;25:1337. doi: 10.1186/s12913-025-13348-7 (PMC12509401; doi:10.1186/s12913-025-13348-7)
Supplement: Supplementary file 2 — Supplementary Material 2: Appendix 2. Redcap data extraction categories [file 12913_2025_13348_MOESM2_ESM.docx]

**Appendix 2:** Redcap data extraction categories

***Background information***

Name of policy source organisation

Date/time range of document

Title of policy

Document purpose (can choose more than one) – is this policy focused on:

- Staff retention
- EDI
- Immigration
- Recruitment
- Other (please specify - free text)

For each of the policy aims listed in sections 1 - 5, say whether the document considers them. Where you answer yes, summarise:

1. The policy aims
2. The policy in relation to ethnicity/race/migrant status. Add any comments on intersecting factors: gender age seniority occupational role
3. Any description of costs
4. Any description of how this policy is being evaluated

***Section 1***

- Promotion and progression
- Recognition and rewards
- Staff training and development

***Section 2***

- Line management and appraisal processes
- Complaint, harassment, bullying and discrimination processes
- Organisational culture/cross-culture
- Disciplinaries and probation
- Organisational leadership (e.g. EDI-balanced)

***Section 3***

- Induction and onboarding
- Visas
- Settling in
- Accommodation
- Special leave for new immigrants
- Schools or daycare or childcare
- Partners
- Language needs

***Section 4***

- Communication needs
- Workload (including shift work, job shares etc related to this, staff numbers, overtime)
- Work roles (including admin load)
- Decision making processes or teamwork (for example team meetings to generate shared decisions) or hierarchies
- Times of cultural importance and needs for these eg Eid and fasting
- Resources such as documents and support for day-to-day tasks
- Mentoring/coaching/buddying schemes
- Antiracism actions such as bystander policy, cultural awareness training

***Section 5***

- Religious needs
- Culturally appropriate occupational health and reasonable adjustments (including disability)
- Culturally appropriate psychological support e.g. counselling
- Culturally appropriate healthcare
- Minoritised staff support networks
- Local amenities
- Leisure activities

***Section 6***

Does the document make only general comments about policy for staff from minoritised ethnic groups/overseas, in ways not already entered as data? If yes, summarise

For each of the following bullet points, say whether the document considers them. Where you answer yes, summarise:

1. The policy aims
2. The policy in relation to ethnicity/race/migrant status. Add any comments on intersecting factors: gender age seniority occupational role
3. Any description of costs
4. Any description of how this policy is being evaluated

- Autonomy
- Increasing feelings of self-competence
- Increasing relatedness
- Increase on-the-job embeddedness
- Increase off-the-job embeddedness
- Reduce job demands
- Increase job resources
- Share and embed best practice

***Final questions***

Is the document accessible for those with for example language or disability needs? Describe

If you wish to make any other comments about the document, please add them here.
