# Supplementary material for: A horizon scanning exercise to explore retention policies for international and minoritised NHS Trust staff in England: what are the current pledges and where are the gaps?
Source: BMC Health Serv Res. 2025 Oct 9;25:1337. doi: 10.1186/s12913-025-13348-7 (PMC12509401; doi:10.1186/s12913-025-13348-7)
Supplement: Supplementary file 3 — Supplementary Material 3. [file 12913_2025_13348_MOESM3_ESM.docx]

[Equality, diversity and inclusion reports - About Us](https://www.somersetft.nhs.uk/about-us/about-us/publication-scheme/2248-2/equality-diversity-and-inclusion-reports/)

[Equality-Delivery-System-EDS2-DCHFT-Report-and-Action-Plan-2024.pdf](https://www.dchft.nhs.uk/wp-content/uploads/2025/02/Equality-Delivery-System-EDS2-DCHFT-Report-and-Action-Plan-2024.pdf)

[Equality Diversity Inclusion 30 Dec 20 FF](https://www.gwh.nhs.uk/media/bqxnxhug/gwhnhsft-equalitydiversityandinclusion-strategy2020-2024.pdf)

[gwhnhsft-peoplestrategy2019-2024-200309.pdf](https://www.gwh.nhs.uk/media/nroh5sgw/gwhnhsft-peoplestrategy2019-2024-200309.pdf)

[ourstrategy_2022-2026.pdf](https://www.salisbury.nhs.uk/media/5gwpjffv/ourstrategy_2022-2026.pdf)

[Microsoft Word - EDI Strategy_v4](https://www.royalsurrey.nhs.uk/download.cfm?doc=docm93jijm4n29339.pdf&ver=73856)

[Microsoft Word - OHFT Strategy 2021-26 BOARD APPROVED](https://www.oxfordhealth.nhs.uk/wp-content/uploads/2021/08/Oxford-Health-NHS-Foundation-Trust-Strategy-2021-26-Full-version-v2.pdf)

[Equality, diversity and inclusion :: NHS Kent and Medway](https://www.kentandmedway.icb.nhs.uk/about-us/access-to-information/equality-diversity-inclusion)

<https://www.surreyandsussex.nhs.uk/application/files/6217/0169/9944/SASH_Annual_Report_v9_final.pdf>

[Our Corporate Strategy to 2025](https://www.berkshirehealthcare.nhs.uk/media/109515089/berkshire-healthcare-corporate-strategy-2023-2025.pdf)

[6590_SABP_Strategy_Doc_2022_CPTs_Alt_v30.pdf](https://www.sabp.nhs.uk/application/files/5516/9944/3861/6590_SABP_Strategy_Doc_2022_CPTs_Alt_v30.pdf)

[MFTEDI Strategy 2019 A4 NO Icons.indd](https://mft.nhs.uk/app/uploads/2024/12/MFT-EDI-Strategy-2019-A4.pdf)

[Liverpool Heart and Chest Hospital | Equality, Inclusion, Diversity and Belonging](https://www.lhch.nhs.uk/equality-inclusion-diversity-and-belonging)

[88e4daad-d524-4eef-945d-c64f5ac3e069.pdf](https://www.stockport.nhs.uk/temparea/88e4daad-d524-4eef-945d-c64f5ac3e069.pdf)

[Our strategy - Essex Partnership University NHS Foundation Trust](https://www.eput.nhs.uk/about/trust/strategy/)

[Our Strategy 2024-2030](https://www.qehkl.nhs.uk/Document/Strategies/our-strategy-2024-2030.pdf)

[Royal Papworth Hospital Strategy 2020-2025](https://royalpapworth.nhs.uk/application/files/8217/3108/3979/146046_RPH_Strategy_2020_25_A4_booklet_FINAL_Spreads.pdf)

[Microsoft Word - Appx 3 Full EDI strategy 2023-30 Trust Board 5.10.23 FINAL](https://www.pah.nhs.uk/download.cfm?doc=docm93jijm4n3078.pdf&ver=3626)

[EDI-Strategy-2024_V10.pdf](https://www.enherts-tr.nhs.uk/wp-content/uploads/2024/04/EDI-Strategy-2024_V10.pdf)

[Belonging and Inclusion Strategy](https://www.hpft.nhs.uk/about-us/equality-and-diversity/belonging-and-inclusion-strategy/)

[Equality, Diversity and Inclusion Annual Report :: Kingston and Richmond NHS Foundation Trust](https://www.kingstonandrichmond.nhs.uk/about-us/equality-diversity-and-inclusion/equality-diversity-and-inclusion-annual-report-kingston-hospital-nhs-foundation-trust-and-hounslow-and-richmond-community-health)

[gesh-five-year-strategy-full-document.pdf](https://www.stgeorges.nhs.uk/wp-content/uploads/2023/05/gesh-five-year-strategy-full-document.pdf)

[Primary Care Mental Health Service](https://www.westlondon.nhs.uk/application/files/8317/0679/5013/Annual_Summary_Equality_Report-2024_v7.pdf)

<https://www.moorfields.nhs.uk/mediaLocal/1vspfpos/strategy-2022-web-version-compressed.pdf>

[oxleasstrategy20212421521pdf-.pdf](https://oxleas.nhs.uk/download/oxleasstrategy20212421521pdf-.pdf?ver=670&doc=docm93jijm4n871)

[PowerPoint Presentation](https://www.homerton.nhs.uk/download/doc/docm93jijm4n13362.pdf?amp;ver=30420)

[Policies and procedures - Website](https://swlstg.nhs.uk/policies-and-procedures?media_item=987&media_type=10#file-viewer)

[ELFT-EDI-annual-report-2023.pdf](https://www.elft.nhs.uk/sites/default/files/2024-10/ELFT-EDI-annual-report-2023.pdf)

[902533_elft_people_plan_strategy_a5_mar22-v6-final-2.pdf](https://i.emlfiles4.com/cmpdoc/4/5/4/5/8/files/902533_elft_people_plan_strategy_a5_mar22-v6-final-2.pdf?dm_i=1TXQ%2C7VJJU%2CQFLSPD%2CW5TMQ%2C1)

[CNWL_Trust_Strategy_2022-2025.pdf](https://www.cnwl.nhs.uk/application/files/6816/8803/5942/CNWL_Trust_Strategy_2022-2025.pdf)

[download.cfm](https://slam.nhs.uk/download.cfm?doc=docm93jijm4n465.pdf&ver=580)

[ML4506_CLCH_Equality_Strategy_Infographic_2021-2025.pdf](https://clch.nhs.uk/application/files/5416/1425/0719/ML4506_CLCH_Equality_Strategy_Infographic_2021-2025.pdf)

[-equality-diversity-and-inclusion-strategy-20222025nbsp.pdf](https://www.northlondonmentalhealth.nhs.uk/download/-equality-diversity-and-inclusion-strategy-20222025nbsp.pdf?ver=7023&doc=docm93jijm4n5083)

[230919-people-and-organisational-development-strategy-finalpdf.pdf](https://www.northlondonmentalhealth.nhs.uk/download/230919-people-and-organisational-development-strategy-finalpdf.pdf?ver=12137&doc=docm93jijm4n2617)

[WRES Action Plan - 2024 Final.pdf](https://www.uhcw.nhs.uk/download/clientfiles/files/WRES%20Action%20Plan%20-%202024%20Final.pdf)

[equality-diversity-and-inclusion-strategy-action-plan-20212024.pdf](https://www.kgh.nhs.uk/download/equality-diversity-and-inclusion-strategy-action-plan-20212024.pdf?ver=10319&doc=docm93jijm4n4626.pdf)

[download.cfm](https://bwc.nhs.uk/download.cfm?doc=docm93jijm4n14350.pdf&ver=22058)

[Equality Diversity and Inclusion Action Plan 2023](https://www.covwarkpt.nhs.uk/download.cfm?doc=docm93jijm4n7343.pdf&ver=9916)

[BCHFT People Strategy 2020-2023](https://www.blackcountryhealthcare.nhs.uk/application/files/2816/2212/5933/BCHFT_People_Strategy.pdf)

[Our-Corporate-Strategy-2022–2027.pdf](https://www.bradfordhospitals.nhs.uk/wp-content/uploads/2022/06/Our-Corporate-Strategy-2022%E2%80%932027.pdf)

[CHFT-strategy-Final_090323Update](https://www.cht.nhs.uk/fileadmin/site_setup/contentUploads/About_us/Publications/Strategies/CHFT-strategy-Final_090323Update.pdf)

[From Ambition to Action](https://www.bdct.nhs.uk/wp-content/uploads/2024/12/Ambition-to-Action-strategy-2023-2026-FINAL.pdf)

[Clatterbridge_Full_5_Year_Strategy_2021-2025_-_FINAL_FOR_WEBSITE.pdf](https://www.clatterbridgecc.nhs.uk/application/files/4417/0600/6062/Clatterbridge_Full_5_Year_Strategy_2021-2025_-_FINAL_FOR_WEBSITE.pdf)

[People_Commitment_FINAL.pdf](https://www.clatterbridgecc.nhs.uk/application/files/2017/0921/3638/People_Commitment_FINAL.pdf)
